# Loss of CPAP causes sustained EGFR signaling and epithelial-mesenchymal transition in oral cancer

**DOI:** 10.18632/oncotarget.27932

**Published:** 2021-04-13

**Authors:** Radhika R. Gudi, Harinarayanan Janakiraman, Philip H. Howe, Viswanathan Palanisamy, Chenthamarakshan Vasu

**Affiliations:** ^1^Department of Microbiology and Immunology, Medical University of South Carolina, Charleston, South Carolina, USA; ^2^Department of Biochemistry, Medical University of South Carolina, Charleston, South Carolina, USA

**Keywords:** tumorigenesis, epithelial-mesenchymal transition, oral squamous cell carcinoma, EGFR, CPAP

## Abstract

Higher epidermal growth factor receptor (EGFR) signaling can contribute to tumor metastasis and resistance to therapies in oral squamous cell carcinoma (OSCC). EGFR signaling can promote epithelial-mesenchymal transition (EMT) in OSCC. EMT is a process by which epithelial cells acquire invasive properties and it can contribute to tumor metastasis. Not only do the abnormal functions of microtubule and microtubule-organizing centers (MTOC) such as centrosomes lead to cancers, but also the malignant tissues are characterized by aberrant centriolar features and amplified centrosomes. Microtubule inhibition therapies increase the sensitivity to EGFR targeting drugs in various cancers. In this study, we show that the loss of expression of a microtubule/tubulin binding protein, centrosomal protein 4.1-associated protein (CPAP), which is critical for centriole biogenesis and normal functioning of the centrosome, caused an increase in the EGFR levels and its signaling and, enhanced the EMT features and invasiveness of OSCC cells. Further, depletion of CPAP enhanced the tumorigenicity of these cells in a xeno-transplant model. Importantly, CPAP loss-associated EMT features and invasiveness of multiple OSCC cells were attenuated upon depletion of EGFR in them. On the other hand, we found that CPAP protein levels were higher in EGF treated OSCC cells as well as in oral cancer tissues, suggesting that the frequently reported aberrant centriolar features of tumors are potentially a consequence, but not the cause, of tumor progression. Overall, our novel observations show that, in addition to its known indispensable role in centrosome biogenesis, CPAP also plays a vital role in suppressing tumorigenesis in OSCC by facilitating EGFR homeostasis.

## INTRODUCTION

Head and neck squamous cell carcinoma (HNSCC) represents the sixth most common cancer with more than 600,000 new patients diagnosed worldwide and it is linked to more than 300,000 deaths every year [1]. Most of the head and neck cancers are squamous cell carcinomas (HNSCC) that arise from mucosal surfaces of the oral cavity (OSCC). In many cancers including OSCC, altered epidermal growth factor receptor (EGFR/ErbB1/HER1) levels contribute to tumorigenesis, metastasis and resistance to therapies, and are linked to poor rate of patient survival [2–6]. The epithelial-mesenchymal transition (EMT), a process by which epithelial cells acquire a mesenchymal and invasive phenotype, contributes to these features [7, 8]. EGFR is significantly altered in OSCC and its prolonged signaling is mitogenic, driving uncontrolled proliferation of tumor cells [9, 10]. In addition, EGFR expression and its signal transduction pathways play an important role in determining the sensitivity to chemo or radiotherapy [11, 12]. Importantly, excessive signaling by this receptor also contributes to EMT in tumor cells, which makes them more invasive and metastatic, and resistant to chemotherapy [13–16]. Hence, this receptor has become one of the major targets for new therapies being investigated in OSCC [13, 17, 18]. Despite these advances in the understanding of EGFR signaling, the regulatory mechanisms underlying EGFR signaling and their effects on cancer initiation, progression and metastasis are not fully understood.

Recent studies have shown that microtubule inhibition causes EGFR inactivation or increases the sensitivity to EGFR targeting drugs in various cancers including OSCC [19–22]. Microtubules and microtubule-organizing center (MTOC) have multiple roles in cellular functions including homeostasis of cell signaling, formation of cilia, cytoskeletal actin organization, and centrosome/centriole duplication and normal cell division [23]. Deregulation in the number of centrioles and centrosomes occurs in cancer and was shown to contribute to tumorigenesis [24–27]. Reports by us [28–30] and others [31–36] have shown a pivotal role for key centriole proteins including the microcephaly-associated protein CPAP (SAS-4; encoded by CENPJ gene) in centriole duplication. Several studies have shown that CPAP, a microtubule binding protein, is indispensable in regulating the centriole and cilia dimensions [28–33, 37–39]. It has also been shown that inhibition of CPAP-tubulin interaction prevents the proliferation of centrosome-amplified cancer cells [40]. Importantly, malignant tissues are characterized by amplified centrioles and other aberrant centrosomal features [25]. However, it is not known if abnormal centrosomal features including aberrantly higher expression of centriolar proteins are the cause or consequence of tumorigenesis.

In this manuscript, we show that CPAP depletion leads to elevated cellular levels of, and signaling by, EGFR in them. CPAP-loss alone is sufficient to cause spontaneous EMT-like features in OSCC cells and increase their invasiveness and tumorigenicity. EGFR depletion suppresses the EMT-features in, as well as the invasiveness of, CPAP-depleted OSCC cells. Paradoxically, however, we show not only that EGFR signaling, which is known to contribute to EMT [41–43], upregulates the cellular levels of CPAP in OSCC cells, but also detected higher CPAP protein levels in OSCC tumors. This suggests that higher centrosome amplification and aberrant centriole features, which are frequently reported phenotypes in cancer [40, 44–46], are possibly the consequence of tumor progression-associated inflammation. Overall, however, our study shows that under normal conditions, CPAP exerts tumor preventive function in OSCC through promoting the homeostasis of EGFR and suppressing EMT.

## RESULTS

### CPAP depletion endows OSCC cells with EMT properties

**Figure 1:**
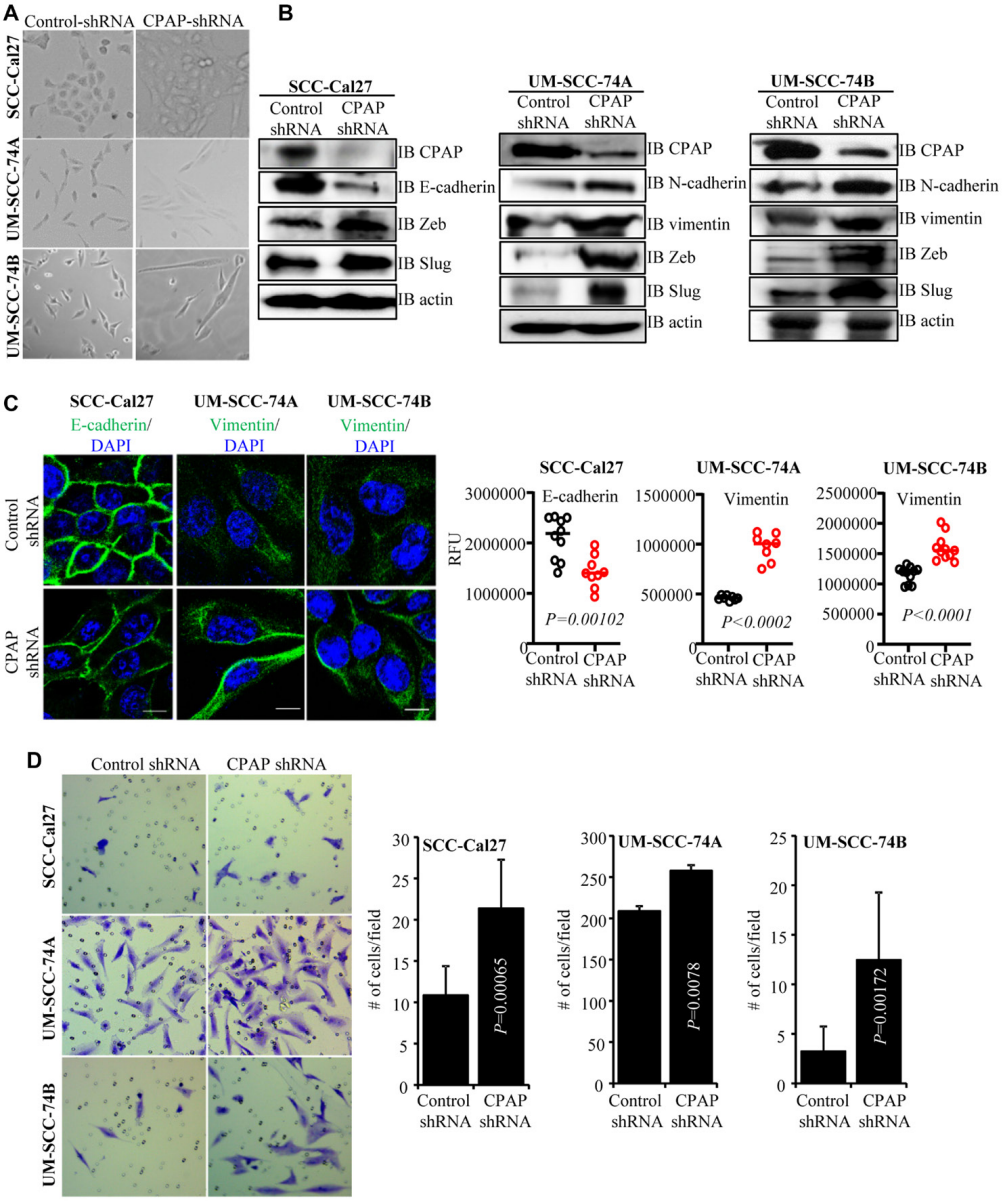
CPAP depleted cells show spontaneous EMT-like morphology and upregulated mesenchymal protein expression. (**A**) Representative bright-field images of indicated oral cancer cell lines transduced with control-shRNA or CPAP-shRNA expressing lentiviral particles and selected using puromycin for 7 days. (**B**) IB showing protein levels of various EMT associated markers along with CPAP and β-actin in indicated cell lines that are stably expressing control-shRNA and CPAP-shRNA. (**C**) Control-shRNA and CPAP-shRNA expressing OSCC cells were fixed and permeabilized, and subjected to immunofluorescence microscopy to detect E-cadherin or vimentin (green) and nuclear stain DAPI (blue). Representative images (left panels) and relative fluorescence intensities (RFUs) of multiple cells/cell areas, quantified using ImageJ application (right panels) are shown. Scale bar: 10 μm. (**D**) Transwell-membrane plates with matrigel coating were used to determine the invasive properties of OSCC cell-lines. Equal numbers of control-shRNA and CPAP-shRNA expressing indicated cell-lines were seeded in serum free media in the upper chamber and incubated for 24 h. Cells on lower chamber side of the transwell membrane were stained with crystal violet, imaged and the average number (mean ± SD) of cells from at least five fields were plotted for each group. Representative results from one of the three independent experiments are shown. All *P*-values are by two-tailed, unpaired Mann-Whitney test.

While conducting centriole biogenesis-related studies using HeLa cells, we observed that depletion of CPAP resulted in a typical EMT-like morphological change (not shown). Since OSCC is a highly prevalent cancer, we examined EMT features of OSCC cells after CPAP depletion. Stable CPAP depletion was performed in 3 oral cancer cell-lines with: 1) an epithelial phenotype (SCC-Cal27), 2) a mesenchymal phenotype of primary tumor origin (UM-SCC-74A), and 3) a mesenchymal phenotype of recurrent tumor origin (UM-SCC-74B). Stable CPAP-depleted and control cell-lines were generated by lentiviral transduction (using pLKO.1-puro vectors encoding CPAP shRNA and scrambled control shRNA) followed by selection using puromycin. As observed in Figure 1A, all three oral cancer cell-lines, with epithelial and mesenchymal phenotypes that were stably expressing CPAP-shRNA showed elongated morphology, a key feature of EMT, compared to control shRNA expressing cells. The spindle-like stretched appearance was more prominent in the CPAP-depleted OSCC cells with mesenchymal properties. IB analysis of these cells revealed the elevated expression of one or more mesenchymal markers (vimentin, N-cadherin, Zeb and Slug) in all three cell types upon CPAP depletion compared to their control counterparts (Figure 1B). Although SCC-Cal27 did not show detectable levels of vimentin, diminished levels of epithelial marker E-cadherin and higher expression of transcription factors Zeb and Slug were detected in these cells upon CPAP depletion. On the other hand, both UM-SCC-74A and UM-SCC-74B cells with CPAP depletion showed higher levels of mesenchymal markers vimentin, N-cadherin, Zeb and Slug. E-cadherin and vimentin protein levels were also examined in control and CPAP-shRNA expressing OSCC cells by immunofluorescence microscopy. As observed in Figure 1C, while CPAP-depleted SCC-Cal27 cells expressed relatively lower levels of E-cadherin, CPAP-depleted UM-SCC-74A and UM-SCC-74B cells expressed higher levels of vimentin compared to respective control cells. In addition to OSCC cells, CPAP-depleted TERT-immortalized non-transformed oral keratinocytes (OKF6) showed lower E-cadherin expression compared to control cells (Supplementary Figure 1A). Conversely, CPAP overexpression diminished the protein levels of EMT or mesenchymal markers in UM-SCC-74B cells and increased the expression levels of E-cadherin in OKF6 cells (Supplementary Figure 1B). These observations suggest that CPAP has a suppressive effect on EMT and loss of CPAP expression renders cells more susceptible to undergo EMT.

Since, cells undergoing the EMT process are known to be more invasive, control-shRNA and CPAP-shRNA expressing cells were subjected to cell migration and invasion assays using trans-well inserts. While the migratory properties of control and CPAP-depleted cells in membrane-well plates without matrigel coating were not different (not shown), all three OSCC cell lines with CPAP depletion showed significantly higher matrigel invasion ability compared to respective control cells (Figure 1D). Collectively, these observations suggest that CPAP plays a tumor suppressive role by inhibiting the EMT process in OSCC cells.

### CPAP loss enhances OSCC cell-induced tumor growth *in vivo*

**Figure 2:**
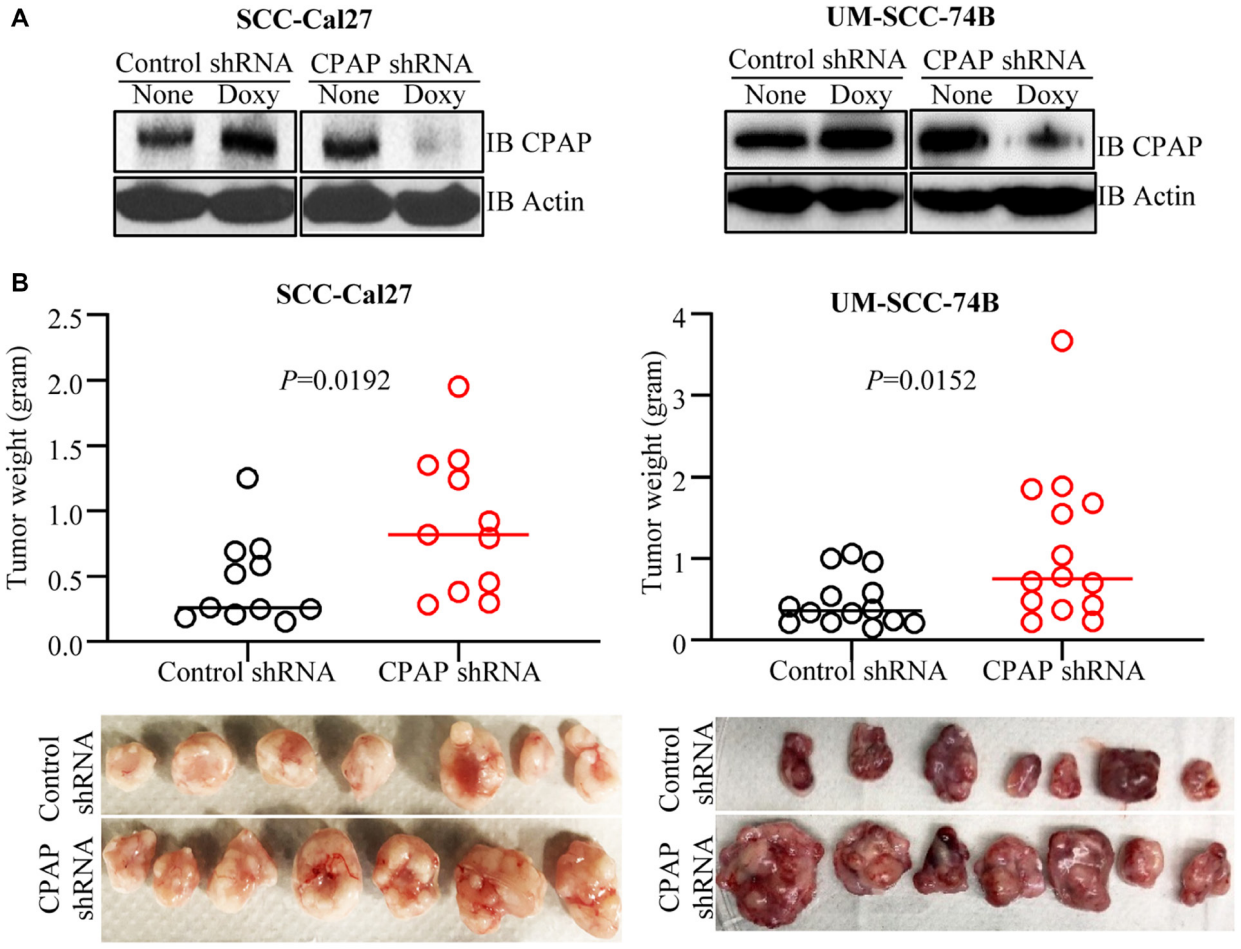
CPAP depletion enhances the tumorigenic properties of OSCC cell lines. SCC-Cal27 and UM-SCC-74B cell lines were transduced with control-shRNA or CPAP-shRNA (under doxy-inducible promoter) lentiviral particles, and the cells with stable integration were selected using puromycin. (**A**) shRNA expression was induced in these cells by culturing for 72 h in the presence of doxy (10 μg/ml) and subjected to IB to detect CPAP and β-actin. (**B**) Eight-week-old nude mice were injected s.c. with these control-shRNA and CPAP-shRNA lentivirus transduced cells (approx: 2 × 10^6^ cell/mouse) on the flank, after mixing with equal volume of matrigel, and monitored for tumor progression. These mice were euthanized after 24 days (UM-SCC-74B cell recipients) or 48 days (SCC-Cal27 cell recipients) to determine the tumor weight. Cumulative data from two independent experiments (total of 11 mice/group for SCC-Cal27 cells and 14 mice/group for UM-SCC-74B cell recipients) are shown (B; upper panels). Images of tumors harvested from one experiment (7 mice/group) are also shown (B; lower panels). *P*-values are by two-tailed, unpaired Mann-Whitney test.

To test if the EMT-like features and enhanced invasiveness of CPAP-depleted OSCC cells can translate into rapid tumor growth *in vivo*, SCC-Cal27 and UM-SCC-74B cells stably expressing control-shRNA and CPAP-shRNA under a doxycycline inducible promoter (Figure 2A) were injected s.c. into the flanks of athymic-nude mice. These mice were given doxycycline (doxy) in drinking water and monitored for tumor growth at timely intervals. Tumor growth was relatively rapid (not shown) in CPAP-shRNA expressing, both SCC-Cal27 (epithelial) and UM-SCC-74B (mesenchymal), cell recipient groups compared to their control-shRNA cell recipient counterparts. Importantly, the average tumor weights upon euthanasia on day 48 (SCC-Cal27 cell recipients) or day 24 (UM-SCC-74B cell recipients) post-injection were significantly higher in CPAP-shRNA expressing cell recipients (Figure 2B). Of note, in our hands, in spite of their mesenchymal phenotype, UM-SCC-74A cells failed to induce tumor in mice even after 50 days post-injection. In addition, control and CPAP-depleted OKF6 cells failed to induce visible tumors in nude mice. In a parallel experiment, a small cohort of mice received s.c. injection of UM-SCC-74B cells that overexpress CPAP under doxy-inducible promoter and were monitored similarly. Tumor weights in mice that received CPAP overexpressing cells were, although not statistically significant, relatively lower compared to control cell recipients (Supplementary Figure 2). Overall, these results along with our *in vitro* findings demonstrating that CPAP knockdown enhances EMT properties and invasiveness (Figure 1) show that CPAP suppresses the tumorigenic properties of OSCC cells.

### CPAP loss enhances total and phospho-EGFR levels in OSCC cells

**Figure 3:**
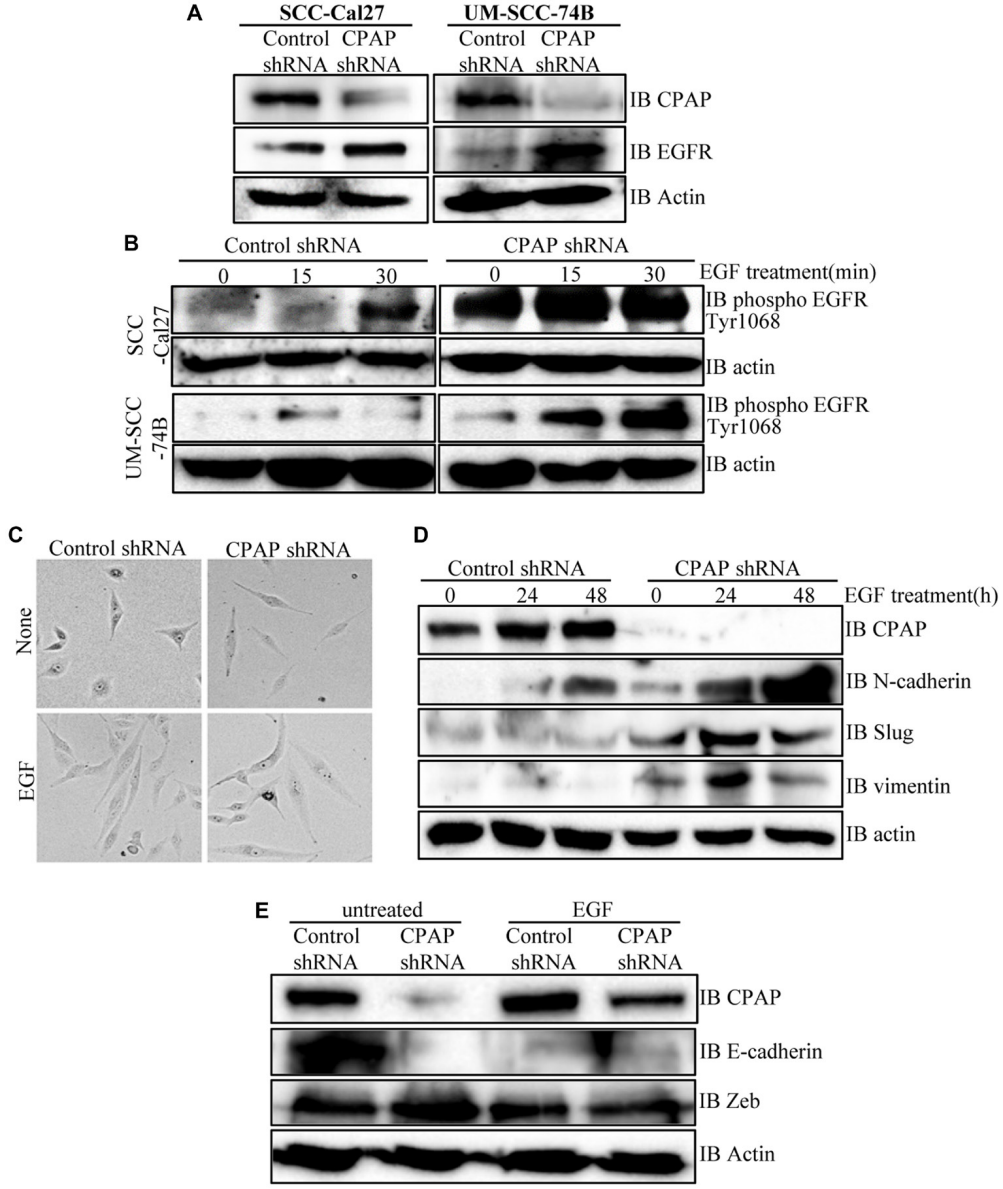
CPAP depletion in OSCC cells causes an increase in cellular levels of total and phospho-EGFR proteins, and EGF treatment enhances EMT-like features in these cells. (**A**) Indicated OSCC cell lines that are stably expressing control shRNA or CPAP shRNA were subjected to IB to detect total EGFR and β-actin proteins. (**B**) These control and CPAP-depleted cell lines were maintained in serum-free media overnight, treated with cycloheximide for 1h and treated with EGF (30 ng/ml), and incubated at 37°C for indicated durations to induce signaling. Levels of phosphorylated EGFR (Tyr1068) were detected in protein equalized cell lysates by IB. (**C**) Representative bright-field microscopy images of control and CPAP depleted UM-SCC-74B cells left untreated or treated with EGF (30 ng/ml) for 48 h are shown. (**D**) IB analysis of control or CPAP-depleted UM-SCC-74B that were left untreated or treated with EGF for indicated durations and subjected to IB to detect the levels of EMT-associated proteins N-cadherin, vimentin and Slug. E-cadherin was undetectable in UM-SCC-74B cells. (**E**) Control and CPAP-depleted SCC-Cal27 cells were also subjected to EGF treatment for 48 h and subjected to IB to detect E-cadherin, Zeb and Slug. Vimentin and N-Cadherin were undetectable in these cells.

EGF treatment is known to promote EMT features in OSCC cells [41, 47]. Hence, our observations that CPAP depletion enhances the mesenchymal features of OSCC cells prompted us to examine EGFR levels in CPAP depleted cells. As shown in Figure 3A and Supplementary Figures 3A and 4A, CPAP depletion resulted in an increase in the cellular EGFR protein levels in OSCC cell-lines with both epithelial (SCC-Cal27 and OKF6) and mesenchymal (UM-SCC-74B and UM-SCC-74A) phenotypes. Reciprocally, CPAP overexpression caused decreased cellular levels of EGFR in both OKF6 and UM-SCC-74B cell-lines (Supplementary Figure 3B). Next, we treated OSCC cells with EGF and examined for the cellular levels of phosphorylated EGFR levels as an indication of the degree of active signaling by this receptor. Figure 3B and Supplementary Figure 4B show that, as expected, EGF treatment resulted in an increase in the levels of phosphorylated EGFR in control OSCC cells. Interestingly, the basal (0 h EGF treatment) levels of phospho-EGFR were profoundly higher in CPAP-depleted cells compared to control cells. Further, the increase in phospho-EGFR levels upon EGF treatment appeared to be more rapid and sustained in CPAP-depleted cells, especially in SCC-Cal27 and UM-SCC-74A cells, compared to control cells. Collectively, these observations suggest that CPAP has a crucial role in maintaining the homeostasis of growth factor receptors like EGFR and loss of CPAP results in, potentially, enhanced and persistent signaling through them.

### EGFR activation under CPAP loss further enhances EMT features of OSCC cells

Since phospho-EGFR levels were higher in CPAP depleted OSCC cells and EGF treatment of these cells caused a rapid increase in this phospho-protein levels, the EMT features of untreated and EGF treated cells were examined. As observed in Figure 3C, UM-SCC-74B cells with and without CPAP knockdown showed the classic spindle-like elongated EMT-associated morphology upon EGF treatment compared to respective control cells. While the untreated CPAP-depleted cells showed spontaneous EMT features (as mentioned in Figure 1A), these morphologic changes were more pronounced upon EGF treatment. Examination of EMT associated markers in these cells revealed a profound upregulation of N-cadherin, but not Slug and Vimentin, levels in control cells upon EGF treatment. However, CPAP depleted cells showed, in addition to higher basal levels compared to control cells, an increase in the levels of N-cadherin, Slug and Vimentin upon EGFR treatment (Figure 3D). Similarly, although the classic EMT associated elongated appearance was not observed with control SCC-Cal27 (epithelial) cells upon EGF treatment alone (not shown), E-cadherin levels were diminished in these control cells upon EGF treatment. However, levels of Zeb, a known EMT modulator, although higher in untreated CPAP-depleted cells as observed in Figure 1, were not higher in either control or CPAP-depleted cells upon EGF treatment (Figure 3E). Overall, these observations support the notion that enhanced EGFR signaling is, at least in part, responsible for the EMT-like features of CPAP-depleted OSCC cells.

### Depletion of EGFR eliminates CPAP-loss associated EMT features of OSCC cells

**Figure 4:**
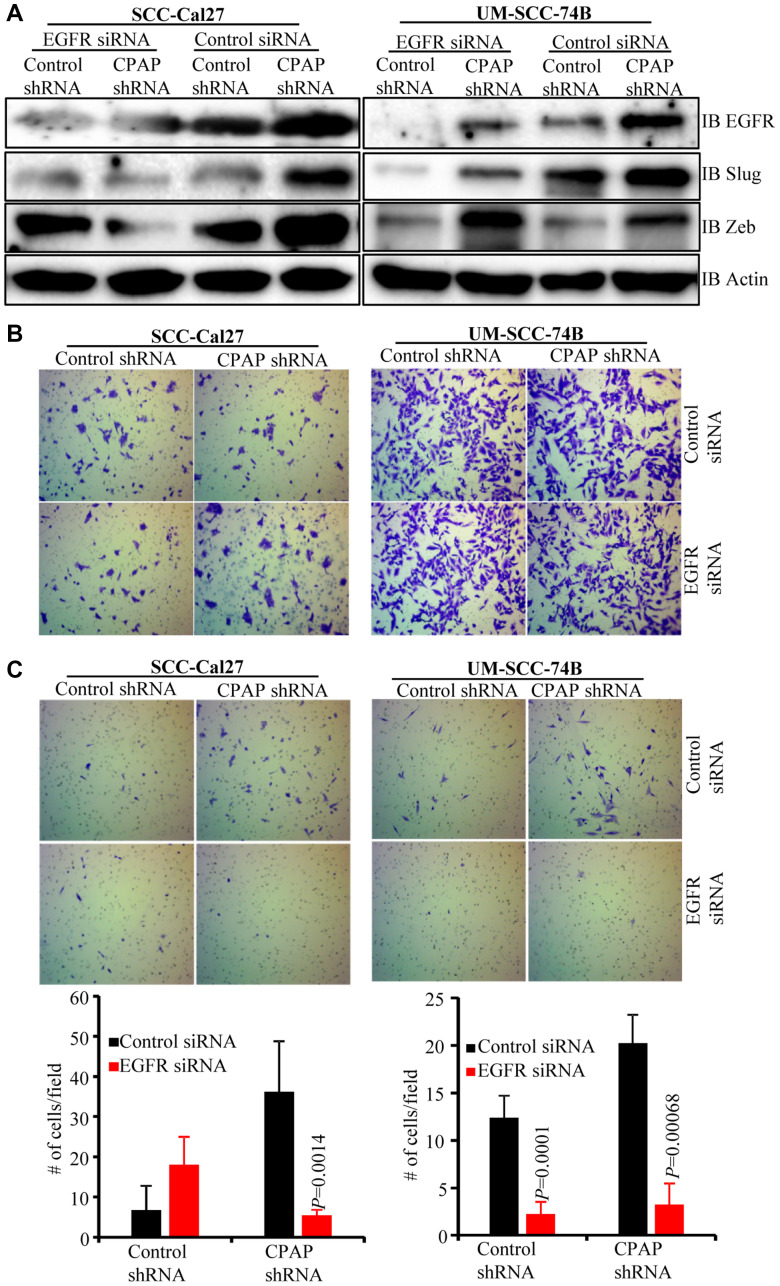
EGFR depletion diminishes the EMT phenotype and invasive property of CPAP-depleted OSCC cells. Control-shRNA and CPAP-shRNA expressing indicated cells were treated with control-siRNA or EGFR-siRNA for 48 h. (**A**) Cells were subjected to IB to detect EMT associated transcription factors Slug and Zeb along with EGFR and β-actin. Equal number of each cell types were also subjected to migration and matrigel-invasion assay using the transwell approach and imaged. (**B**) Representative fields of migrated cells on insert membranes with no matrigel coating are shown. (**C**) Representative fields of invasive cells on membranes with matrigel coating (upper panels) and average number (mean ± SD) of cells of at least 5 fields/group (lower panels) are shown. Representative results from one of the three independent experiments are shown. *P*-values are by two-tailed, unpaired Mann-Whitney test.

To assess the role of EGFR in CPAP-depletion associated EMT features, control and CPAP-depleted SCC-Cal27 and UM-SCC-74B cells were treated with scrambled control or EGFR specific siRNA. As shown in Figure 4A, EGFR depletion in control SCC-Cal27 cells did not affect, or caused only a modest increase in, the expression levels of transcription factors Slug and Zeb compared to control siRNA treated cells. In control UM-SCC-74B cells, EGFR depletion caused suppression of protein levels of Slug, but not Zeb. Interestingly, under CPAP deficiency, EGFR depletion caused the suppression of Zeb and Slug levels in SCC-Cal27 cells and Slug in UM-SCC-74B cells. On the other hand, EGFR depletion under CPAP deficiency caused diminished expression of Zeb by UM-SCC-74A cells (Supplementary Figure 4C). We then examined if these differential changes in EMT marker levels upon EGFR depletion in CPAP depleted cells impacts their migratory and invasive properties. Cells were plated in trans-wells systems without and with matrigel coating to determine the migratory and invasive properties. OSCC cell lines with both epithelial (SCC-Cal27) and mesenchymal (UM-SCC-74B and UM-SCC-74A) phenotypes showed comparable migratory properties, based on the assay using trans-wells without matrigel, irrespective of CPAP and/or EGFR deficiency (Figure 4B and Supplementary Figure 4D). However, while CPAP deficiency, as observed in Figure 1D, enhanced the matrigel invasiveness of all three OSCC cell-lines, CPAP-deficient OSCC cells with EGFR depletion showed profoundly suppressed invasiveness compared to their control counterparts with no EGFR depletion (Figure 4C and Supplementary Figure 4E). Overall, these observations confirm that CPAP-loss associated EMT phenotype, and potentially the enhanced tumorigenic property, of OSCC cells are EGFR-, and its enhanced signaling, -dependent.

### CPAP protein levels in OSCC cell-lines under EMT inducing conditions and steady state

**Figure 5:**
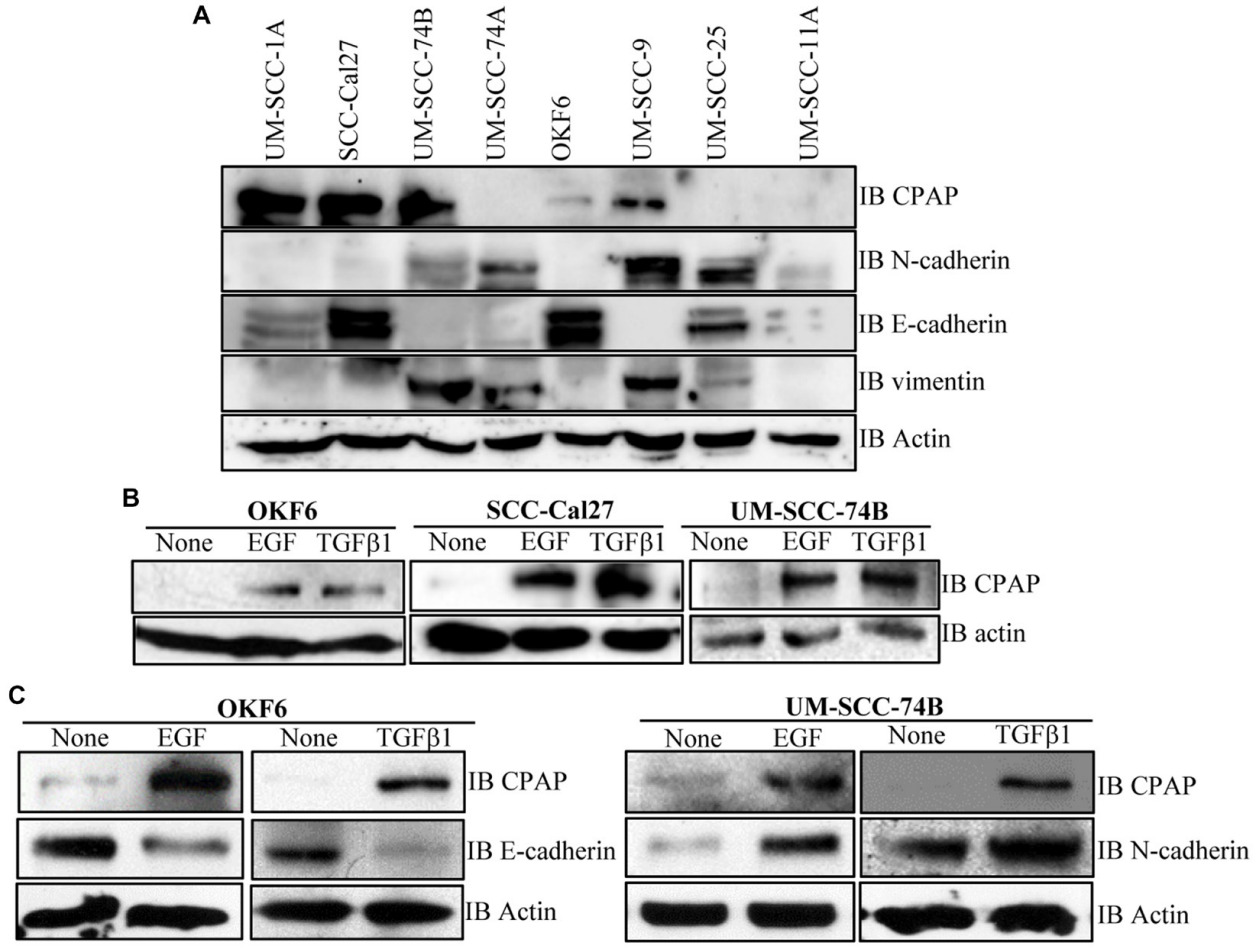
CPAP and EMT marker levels in OSCC and normal cell-lines. (**A**) IB showing protein levels of CPAP, the EMT markers N-cadherin, E-cadherin and vimentin, and β-actin in protein equalized lysates of indicated cell-lines. (**B**) IB showing CPAP and β-actin protein levels in the indicated OSCC cell-lines (SCC-Cal27 and UM-SCC-74B) and transformed oral normal cell-line (OKF6) upon treatment with EGF (30 ng/ml) and TGFβ1 (5 ng/ml) for 48 h. (**C**) IB showing EMT marker E-cadherin or N-cadherin level, along with CPAP and β-actin levels, in OKF6 and UM-SCC-74B OSCC cells.

To determine if there is a correlation between CPAP protein levels and the phenotype of OSCC cells, we examined the basal expression levels of CPAP and EMT associated markers in multiple OSCC cell-lines and normal human oral keratinocyte OKF6. As observed in Figure 5A, when protein equalized cell lysates were employed, UM-SCC-25, and SCC-Cal27 along with immortalized normal OKF6 cells showed higher expression of epithelial marker E-cadherin. However, UM-SCC-74A, UM-SCC-74B and UM-SCC-9 expressed higher levels of mesenchymal markers vimentin and N-cadherin. While UM-SCC-25 cells expressed higher levels of both epithelial and mesenchymal markers, UM-SCC-11A expressed these markers at very low levels. Importantly, however, CPAP protein levels did not show a clear correlation with the EMT marker levels in these cell lines with different phenotypic properties.

Next, we determined the impact of exposure to growth factors that are known to promote EMT and tumorigenesis [47–51] on cellular levels of CPAP. Normal OKF6 cells and OSCC cells, UM-SCC-Cal27 and UM-SCC-74B, were treated with EGF and TGFβ1 and assessed for the CPAP protein levels. As observed in Figure 5B, all three cell lines expressed higher levels of CPAP upon EGF and TGFβ1 treatments. Further, as anticipated EGF and TGFβ1 treatments not only decreased the levels of epithelial marker E-cadherin in OKF6 cells, but also caused an increase in the levels of mesenchymal marker N-cadherin in UM-SCC-74B (Figure 5C). These observations suggest that while CPAP protein levels could be dependent on cell-lines and their various other properties, EMT inducing, and perhaps tumor promoting, inflammatory conditions may contribute to the cellular accumulation of this protein.

### CPAP expression levels correlate with HNSCC tumor grades

**Figure 6:**
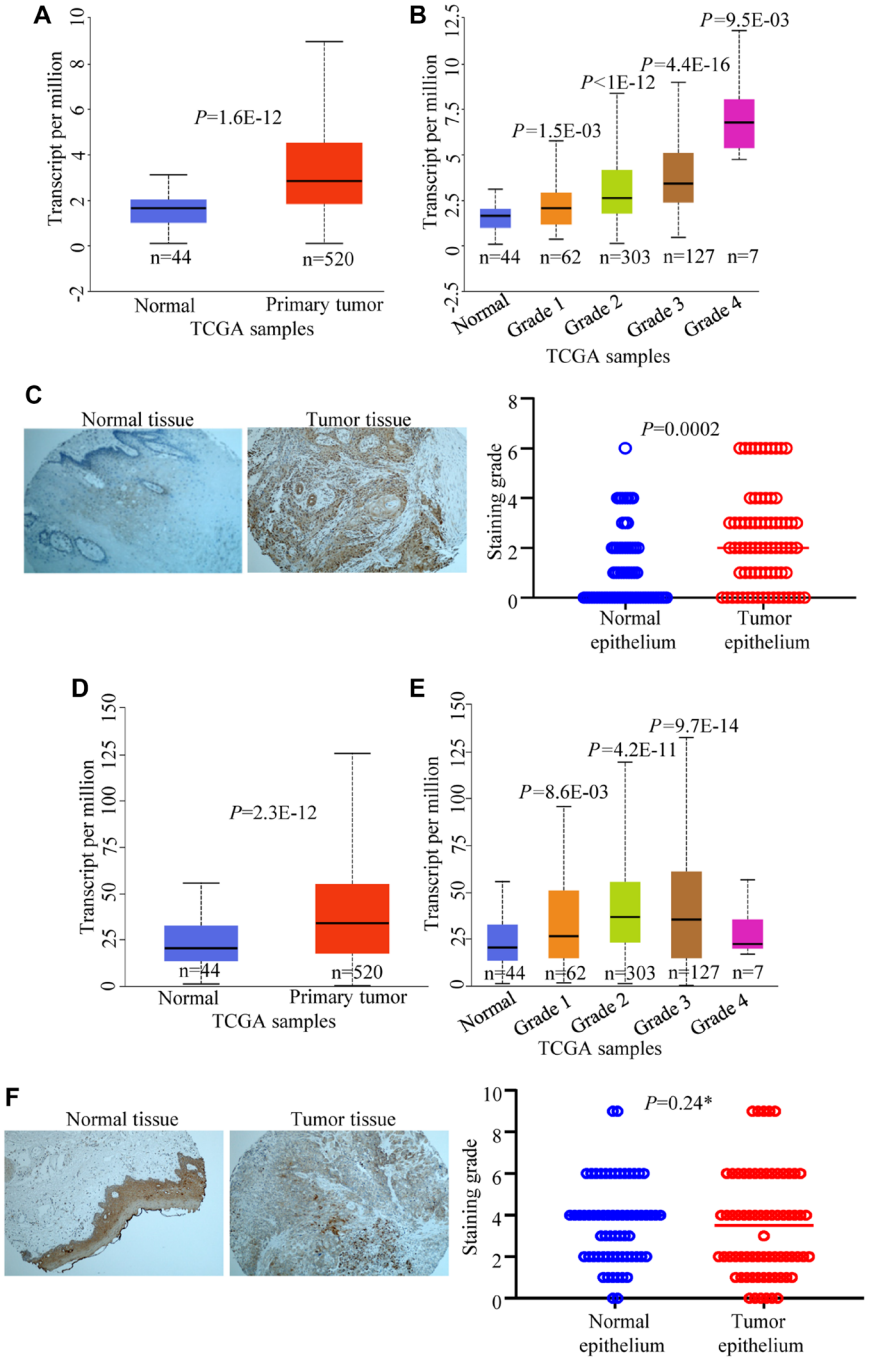
CPAP and EGFR expression levels in HNSCC and normal tissues. TCGA data set was analyzed using UALCAN for CPAP mRNA expression and comparisons were made between head and neck normal tissues with primary tumor tissues (**A**) and different tumor grade subgroups (**B**). Immunohistochemistry of head and neck cancer TMA slides containing tumor tissue and adjacent normal tissue sections was carried out for CPAP protein expression using anti-CPAP antibody (**C**). Images of staining examples of normal and tumor tissues (left) and mean staining intensity grades of tumor and adjacent normal epithelia (right) are shown. TCGA data set was also analyzed for EGFR mRNA expression and comparisons were made between normal tissues with primary tumor tissues (**D**) and different tumor grade subgroups (**E**). TMA staining was performed using anti-EGFR antibody (**F**) and the images of staining examples (left) and mean staining intensity grades (right) are shown. All *P*-values are by two-tailed, unpaired Student *t-test* (normal vs primary tumor for panels A and D; normal vs specific grade for panels B and E; normal epithelium vs tumor epithelium for panels C and F). ^*^denotes not significant.

Since we found that pro-EMT and tumorigenic stimuli such as EGF and TGF can cause cellular accumulation of CPAP in OSCC cells, CPAP expression profiles of tumor and normal tissues of HNSCC patients were probed by examining the public data as well as by performing tissue microarray (TMA) analysis. The Cancer Genome Atlas (TCGA) query revealed that not only significantly higher CENPJ transcript levels are found in the human HNSCC samples (Figure 6A), but also there appears to be a strong positive correlation between CENPJ transcript levels and the HNSCC tumor grades (Figure 6B). We, then, examined the CPAP protein expression pattern in tumor (*n* = 71) and adjacent normal tissues (*n* = 65) of HNSCC patients by TMA analysis. Tumor and adjacent normal tissues were from oral cavity, tongue and oropharynx of HNSCC patients. As observed in Figure 6C, we found that CPAP protein levels were higher in tumor epithelium of HNSCC patients compared to that of normal epithelium. On the other hand, while the EGFR transcript levels were relatively higher in tumor tissues (Figure 6D and 6E), TMA analysis showed no statistically significant difference in the EGFR specific staining levels between HNSCC tumor and adjacent normal tissues (Figure 6F). Overall, these observations, along with the lack of correlation between the levels of CPAP and EGFR in OSCC cell lines (Figure 5), suggest that tumor associated inflammation drives cellular accumulation of CPAP which may not be functional in terms of impacting EGFR signaling, EMT and/or tumor suppression.

## DISCUSSION

Here, using the OSCC model, we demonstrate a negative regulatory role for CPAP, an essential centriole biogenesis protein [52], in tumor prevention by keeping the EGFR signaling regulated and EMT at bay. CPAP is a microtubule and α-tubulin binding protein and the tubulin-binding property [37, 53, 54] is important for its function on the centrioles, especially in restricting the centriole length [32, 33, 55]. In addition, CPAP is required for spindle orientation, which defines normal and asymmetric cell divisions, abnormalities in which could lead to various clinical conditions including tumor malignancies [52, 55–57]. Recently, it has been shown that inhibition of CPAP-tubulin interaction prevents proliferation of centrosome-amplified cancer cells [40]. However, a role for CPAP as a tumor suppressor in regulating EGFR homeostasis and signaling, and EMT is not known. In this report, we show that although EGFR treated OSCC cells as well as HNSCC tissues show higher levels of CPAP protein, loss of CPAP in OSCC cell-lines not only increased their total and phosphorylated EGFR levels, but also enhanced the EMT and tumorigenic properties. Our results shed light on a novel mechanism of tumor suppression by the centriole-associated protein CPAP.

EGFR overexpression was detected in majority of OSCC and many other malignancies [58, 59]. More than 90% of OSCC overexpress EGFR [60, 61]. Associations have been made between the higher expression levels of EGFR and an aggressive phenotype, poor prognosis and resistance to anticancer therapy of OSCC [58]. Several EGFR targeted therapies have been developed for the treatment of advanced OSCC [62, 63]. Several studies have demonstrated that EGF can induce EMT, a process by which epithelial cells adopt a mesenchymal phenotype or fibroblast-like properties and increase the invasiveness, in cancer cells, eventually leading to metastasis [41, 47, 50]. In general, cells undergoing EMT exhibit down-regulation of the epithelial marker E-cadherin and up-regulation of mesenchymal markers such as N-cadherin and vimentin and the transcription factors Zeb, Slug, Snail. Further, cancer cells undergoing EMT switch from their epithelial morphology and characteristics such as non-motile and non-invasiveness to their mesenchymal elongated, motile, and invasive characteristics [64]. Our observations show that loss of CPAP in OSCC cells not only increases the EGFR levels and signaling, as indicated by phospho-protein levels, but also this effect on EGFR endows the cells with EMT-like features, enhanced invasiveness and tumorigenesis.

It has been shown that not only does EGF signaling promote EMT in OSCC cells [41, 47], but also that the cancer cells which are undergoing EMT express considerably lower levels of EGFR and are less susceptible to EGFR targeted therapies, which leads to their chemotherapeutic resistance [65]. Interestingly, while CPAP depletion associated increase in total EGFR appears to show similar trend in different OSCC cell lines, the dynamics of phosphorylated EGFR levels upon ligand activation appear to be different. Basal level of phospho-EGFR upon CPAP depletion was found to be profoundly higher in mesenchymal cell-line UM-SCC-74B which was derived from a recurrent metastatic tumor and in an OSCC cell-line with epithelial phenotype (SCC-Cal27). However, while EGF activation caused rapid downregulation of phospho-EGFR in UM-SCC-74B cells, ligand engagement increases phospho-EGFR levels in CPAP depleted SCC-Cal27. These observations suggest that rapid tumor inducing property of UM-SCC-74B could be due to low threshold activation and persistent signaling of EGFR in these cells, especially under CPAP deficiency. Nevertheless, the common features of CPAP depleted OSCC cells are higher and persistent EGFR function, enhanced invasiveness and/or tumorigenic ability. Importantly, results from our EGFR depletion studies show that these CPAP-deficiency associated effects are, in fact, EGFR- and, perhaps, its persistent signaling-dependent.

Centriole amplification and higher expression of centrosomal proteins are key features of many cancers [40, 44–46]. However, whether these features are the cause of OSCC and other cancers, or a consequence of tumor progression associated events is largely unknown. Tumor microenvironment is inflammatory and known to express higher levels of growth and cell transformation factors such as TGFβ1 and EGF, and/or their receptors [66–70]. In fact, our results show that EGF and TGF treatments, which are known to promote the EMT and tumorigenic potential of cells, causes the accumulation of CPAP protein in cancer cells. Furthermore, detection of significantly higher levels of CPAP protein in HNSCC tissues compared to normal tissues, and lack of correlation of CPAP levels with EGFR expression, suggests that tumor associated inflammation contributes to cellular accumulation of potentially non-functional CPAP. While functional impacts of CPAP accumulation under tumor progression and inflammation need to be investigated in the future, our observations that loss of CPAP in OSCC cells causes increased EGFR levels and signaling, and enhanced EMT and tumorigenic properties suggest that this microtubule/tubulin interacting protein negatively regulates the EMT process, perhaps by promoting EGFR homeostasis. Overall, our observations, reveal that CPAP, a tubulin interacting protein which is critical for centriole biogenesis, has a preventive role in EMT and tumorigenesis by promoting EGFR homeostasis and diminishing the EGFR signaling.

Importantly, when ligands are bound, EGFR is phosphorylated, promoting downstream signal transduction, following which it is internalized, ubiquitinated and degraded in the lysosomes, or recycled back on to cell surface [71, 72]. These events involve microtubules and endocytic vesicular transport pathways [9, 73, 74]. Previous reports show that dysfunction of trafficking of EGFR to lysosome causes abnormal enlarged endosomes that prevent the timely degradation of EGFR, resulting in its persistent signaling and profoundly enhancing cell proliferation [75–80]. Therefore, considering the role of microtubules in the transport of ligand engaged EGFR, we believe that CPAP regulates this homeostatic mechanism of EGFR. Therefore, it is possible that CPAP may not be directly involved in the EGFR signaling pathway, but it facilitates the EGFR degradative pathway post-ligand engagement. This aspect needs to be investigated in the future.

While it has been reported before that EGF stimulation endows OSCC cells with stem cell-like properties, increased invasiveness, and tumorigenic properties [41, 47], the molecular mechanisms underlying the regulation of EGF induced EMT and tumorigenicity were not known. Hence, our study does begin to shed light on the molecular mechanisms by which centrosome/MTOC associated proteins are involved in preventing tumorigenesis. Nevertheless, additional studies are needed in the future to address the mechanism by which CPAP suppresses EGFR dependent EMT and tumorigenesis.

## MATERIALS AND METHODS

### Cell culture

Oral cancer cell lines were acquired from ATCC or obtained from the laboratory of Dr. Thomas Carey, University of Michigan. Telomerase reverse transcriptase (TERT)-immortalized human normal oral keratinocyte OKF6tert1 (OKF6) cell line was obtained from Dr. Jim Rheinwald, Harvard Medical School. Most studies used SCC-Cal27 (adenosquamous carcinoma) cell line which possesses epithelial phenotype and UM-SCC-74A and UM-SCC-74B which have mesenchymal features. These cells were cultured in Dulbecco’s modified Eagle medium that was supplemented with 10% fetal bovine serum and other additives such glutamine, sodium pyruvate, bicarbonate, minimum essential amino acids and antibiotics/antimycotic. Cellular CPAP levels were also tested in other oral cancer cell lines such as UM-SCC1A, UM-SCC9, UM-SCC4, UM-SCC25, UM-SCC11A, some of which were cultured in DMEM/F12 medium containing hydrocortisone and above-mentioned supplements. OKF6 cells were cultured in keratinocyte-specific serum free medium (ThermoFisher) supplemented with human recombinant epidermal growth factor and bovine pituitary extract. Cells were cultured in a 37°C incubator with 5% CO_2_.

### Constructs, transfection and generation of stable cell lines

CPAP-depleted stable oral cancer cells were generated using the pLKO.1-TRC-puromycin lentiviral vector-based system (Sigma Mission shRNA system). Constitutive and doxycycline inducible vectors expressing scrambled control-shRNA or CPAP-specific-shRNA (GCTAGATTTACTAATGCCA) showing validated 80% CPAP knockdown were either purchased from the Sigma Mission shRNA library or custom cloned by GenScript. EGFR specific (catalog# sc-29301), and scrambled control siRNAs were purchased from Santa Cruz Biotech. Doxycycline was purchased from Fisher Scientific. Transfections were performed using the TransIT-X2 reagent or TransIT-siquest (MirusBio).

### Antibodies

Commercial available antibodies against CPAP/CENPJ (Proteintech; cat# 11517-1-AP), Actin-HRP (Proteintech; cat# HRP-600008), Zeb (Proteintech; cat# 21544-1-AP), Slug (Proteintech; cat# 12129-1-AP ), N-cadherin (Proteintech; cat# 22018-1-AP ), E-cadherin (Proteintech; cat#20874-1-AP ), vimentin (Cell Signaling Technology; cat#5741), EGFR (Santa Cruz Biotech; cat# sc-373746), phospho-EGFR (Cell signaling Technology; cat# 2234) and GFP (Proteintech; cat# 50430-2-AP) were used. Secondary HRP-linked anti-mouse and -rabbit antibodies were purchased from Biorad and Amersham respectively and Alexa-conjugated antibodies were from Invitrogen.

### Cell lysis and Western blotting

Whole cell lysates were prepared using the RIPA lysis buffer containing protease inhibitors, centrifuged, and supernatants were used for further analysis. Lysates were then subjected to immunoblotting (IB) after separation by SDS- polyacrylamide gel electrophoresis (PAGE) and Western blotting (WB) transfer onto PVDF membrane (Biorad).

### Immunofluorescence

Cells were grown on coverslips and fixed with ice-cold methanol at –20°C for 10 mins. Blocking with 1% BSA as well as primary and secondary antibody dilutions were made in permeabilization buffer and incubations were done at 37°C. Images were acquired using the Zeiss 880 confocal microscope. 63X oil immersion objective with n.a. 1.4 was used in most imaging experiments.

### Migration and invasion assay

Oral cancer cells treated differently were trypsinized, counted and an equal number were seeded in serum free media in the upper trans-well chamber with or without matrigel (filter pore size 8 μm, BD). 5% FBS-containing media was loaded in the bottom chamber as chemoattractant. After 24 h incubation at 37°C, cells on the reverse side of filter were fixed with 2% glutaraldehyde. Cells on the seeded membrane side were scraped off using a wet cotton swab. Filter membranes were then stained with 2% crystal violet, washed, dried, and mounted on glass slides in immersion oil. Multiple areas of the membranes were imaged using a light microscope and average number of cells, counted from the images of multiple fields of membranes from 3 independent experiments, were determined.

### Tumor microarray analysis (TMA) of patient tissues

HNSCC-TMAs containing tumor and adjacent normal tissues from oral cavity, tongue and oropharynx of HNSCC patients were obtained from the Biorepository & Tissue Analysis at Hollings Cancer Centre, subjected to IHC staining using anti-CPAP (Proteintech; cat#11517-1-AP) and anti-EGFR (Santa Cruz Biotech; clone A-10; cat# sc-373746) antibodies, and staining intensities were scored blindly and imaged by the IHC core.

### Tumor xenografts

All animal experiments were approved by the Medical University of South Carolina Institutional Animal Care and Use Committee (IACUC). Eight-week-old male and female athymic nude mice were injected subcutaneously with SCC-Cal27 or UM-SCC-74B cells (2 × 10^6^ cells/mouse) that are stably expressing either control-shRNA or CPAP-shRNA under a doxycycline (doxy)-inducible promoter. The following groups were included: control-shRNA SCC-Cal27 (*n* = 11), CPAP-shRNA SCC-Cal27 (*n* = 11), control-shRNA UM-SCC-74B (*n* = 14), and CPAP-shRNA UM-SCC-74B (*n* = 14). Cell pellets were suspended in 50% matrigel prior to injection. The mice were monitored for tumor growth for up to 24 days (UM-SCC-74B cell recipients) or 48 days (SCC-Cal27 recipients), euthanized at the end of monitoring period, and tumor tissues were excised and weighed. Our pilot studies showed that considerable number of UM-SCC-74B and SCC-Cal27 cell injected mice, CPAP-shRNA expressing cell recipients particularly, reached the experimental endpoint with respect to IACUC approved maximum tumor size of within 24 and 48 -days post-cell transfer, respectively. Therefore, all experiments using these mice were terminated at indicated time-points to assess the tumor mass. In some experiments, OKF6 and UM-SCC-74A that are stably expressing control-shRNA or CPAP-shRNA were injected into nude mice. However, these cells failed to induce visible tumors; hence the data is not shown.

### Statistical analysis

Mean, SD, and statistical significance (*p*-value) were calculated using GraphPad Prism and Microsoft Excel. Unpaired, two-tailed *t-test* or *P* Mann-Whitney test was employed, unless specified in the figure legend, for comparing two groups. A *p* value of ≤ 0.05 was considered statistically significant.

## SUPPLEMENTARY MATERIALS


