# Supplementary material for: Loss of CPAP causes sustained EGFR signaling and epithelial-mesenchymal transition in oral cancer
Source: Oncotarget. 2021 Apr 13;12(8):807–22. doi: 10.18632/oncotarget.27932 (PMC8057274; doi:10.18632/oncotarget.27932)
Supplement: Supplementary file 1 [file oncotarget-12-807-s001.pdf]

Loss of CPAP causes sustained EGFR signaling and epithelial-mesenchymal transition in oral cancer

SUPPLEMENTARY MATERIALS

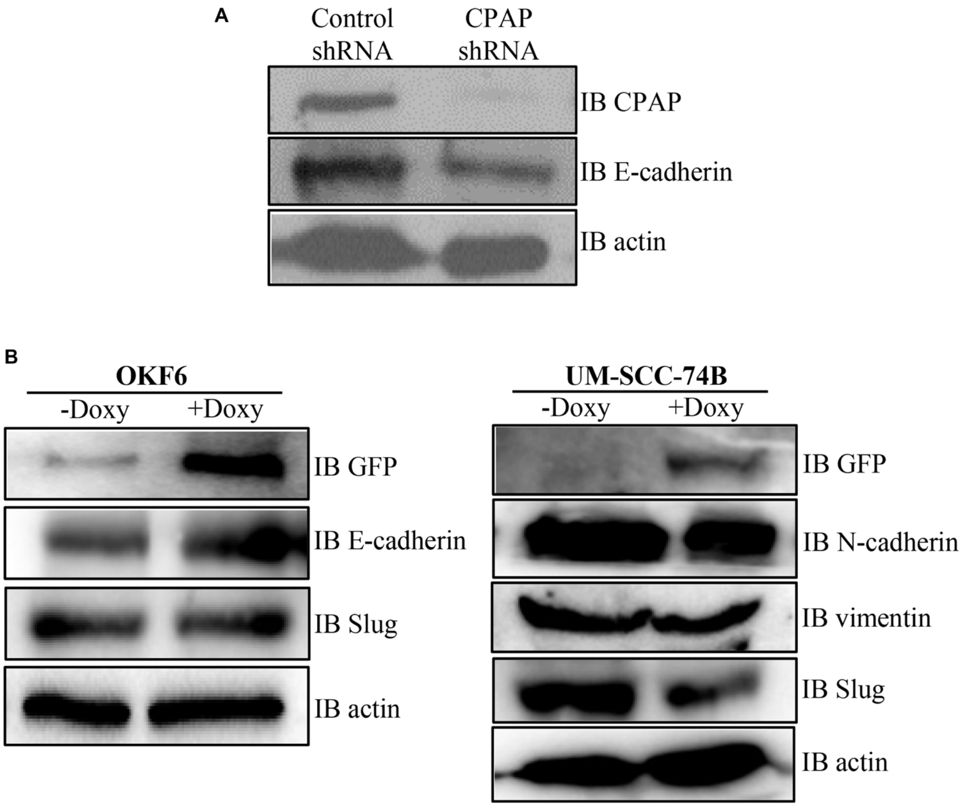

Supplementary Figure 1: EMT marker expression levels in CPAP-depleted and -overexpressing cells. (**A**) IB showing protein levels of E-cadherin along with CPAP and β-actin in OKF6 cells that are stably expressing control-shRNA and CPAP-shRNA. (**B**) OKF6 and UM-SCC-74B cells that are stably expressing GFP-CPAP (under doxy-inducible promoter) vector were left untreated (-doxy) or exposed to doxycycline (+doxy) for 24 h and subjected to IB for detecting various EMT markers along with GFP (CPAP) and β-actin.

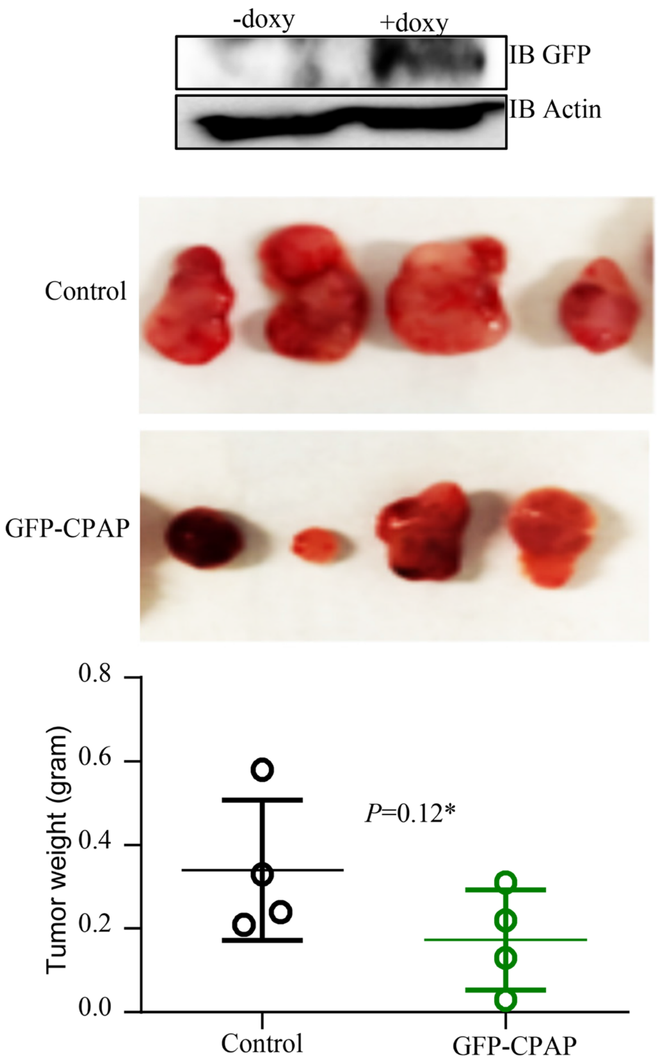

Supplementary Figure 2: CPAP overexpression enhances tumorigenic property of OSCC cells. UM-SCC-74B cells stably expressing control vector or GFP-CPAP (under doxy-inducible promoter) vector or control vector were generated. GFP expression/CPAP overexpression after 24 h post doxy treatment was confirmed by IB of untreated and doxy-treated GFP-CPAP cells (upper panel). Control and GFP-CPAP cells were s.c. injected to the flank of nude mice (4 mice/group). Day 24 post-injection, tumors were harvested and weighed. Tumors (middle panel) and mean ± SD of tumor weights (lower panel) are shown. *P*-value by two-tailed, unpaired Mann-Whitney test. *denotes not significant.

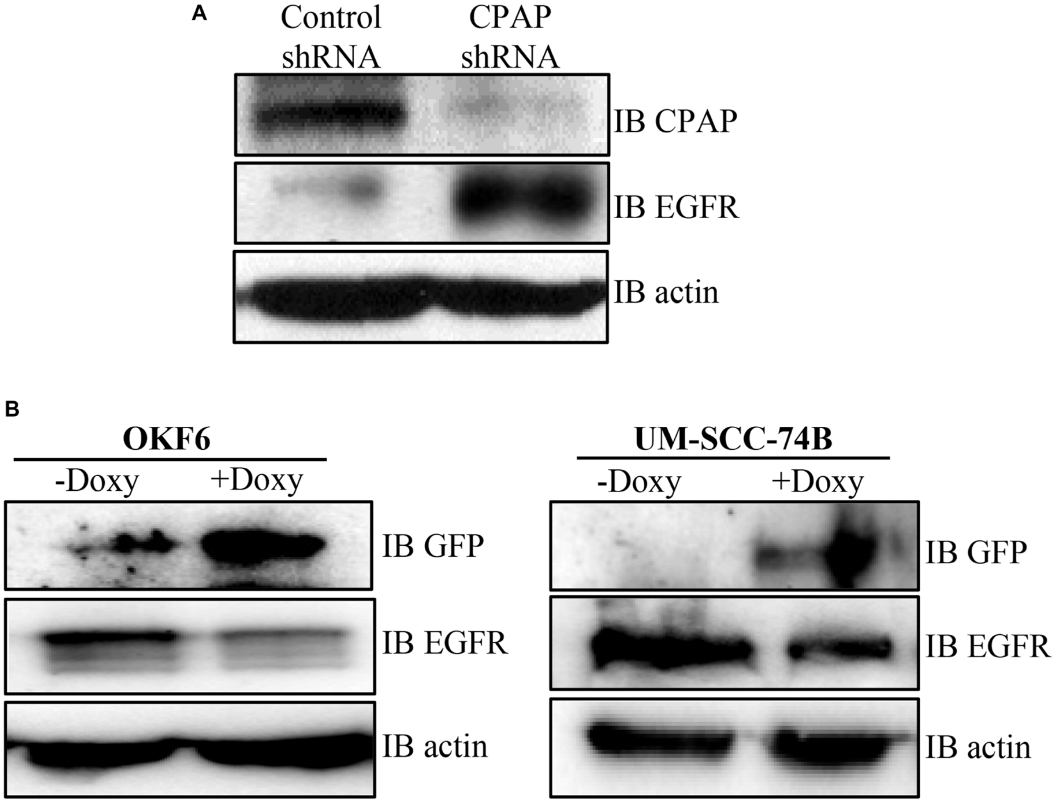

Supplementary Figure 3: EGFR expression levels in CPAP-depleted and -overexpressing cells. (**A**) IB showing protein levels of EGFR along with CPAP and β-actin in OKF6 cells that are stably expressing control-shRNA and CPAP-shRNA. (**B**) OKF6 and UM-SCC-74B cells that are stably expressing GFP-CPAP (under doxy-inducible promoter) vector were left untreated (-doxy) or exposed to doxycycline (+doxy) for 24 h and subjected to IB for detecting EGFR along with GFP (CPAP) and β-actin.

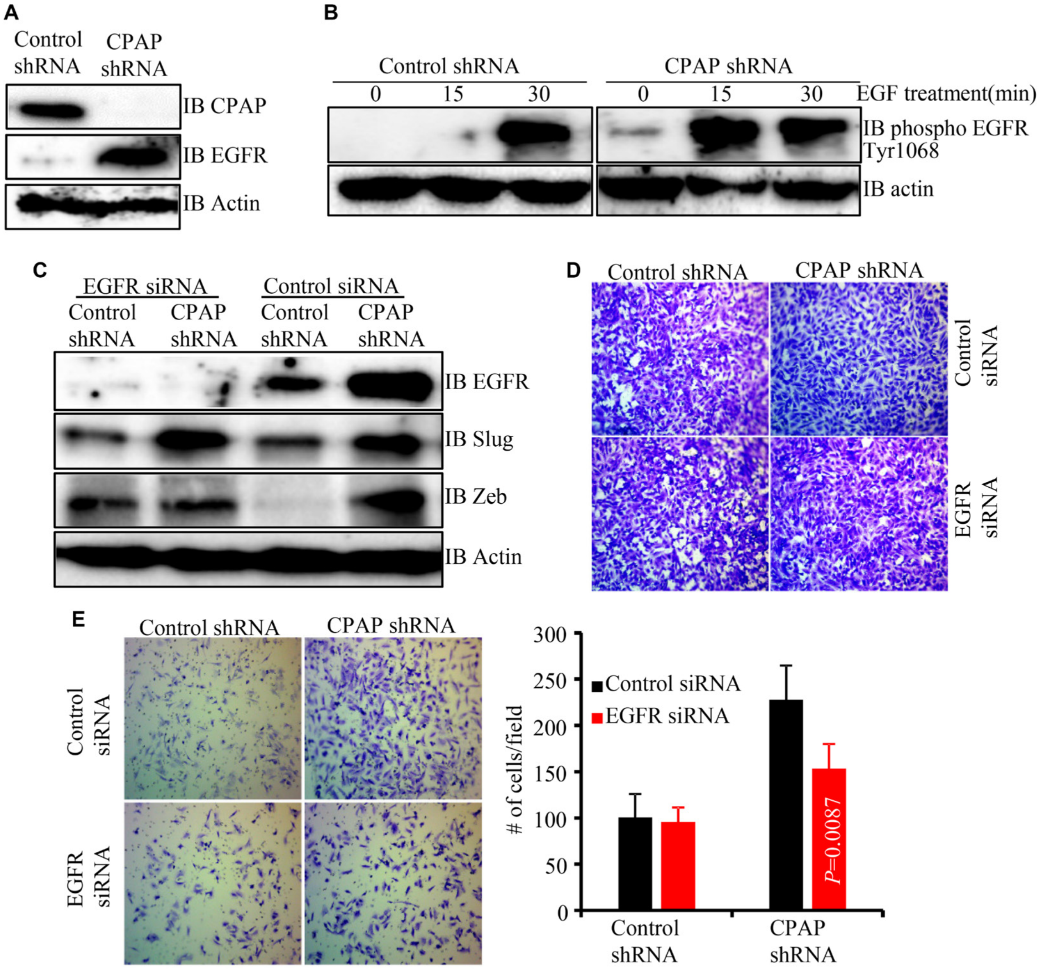

Supplementary Figure 4: CPAP depletion causes an increase in cellular levels of total and phospho-EGFR proteins, and EGF treatment enhances EMT-like features, in UM-SCC-74A cells. (**A**) UM-SCC-74A cells that are stably expressing control shRNA or CPAP shRNA were subjected to IB to detect total EGFR and β-actin proteins. (**B**) These cells were maintained in serum-free media overnight, treated with cycloheximide for 1 h and treated with EGF (30 ng/ml), and incubated at 37°C for indicated durations to induce signaling. Levels of phosphorylated EGFR (Tyr1068) were detected in protein equalized cell lysates by IB. (**C**) Cells were subjected to IB to detect EMT associated transcription factors Slug and Zeb along with EGFR and β-actin. Equal number of each cell types were also subjected to migration and matrigel-invasion assay using the transwell approach and imaged. (**D**) Representative fields of migrated cells on insert membranes with no matrigel coating are shown. (**E**) Representative fields of invasive cells on membranes with matrigel coating (left) and average number (mean ± SD) of cells of at least 5 fields/group (right) are shown. Representative results from one of the three independent experiments are shown. *P*-values by two-tailed, unpaired Mann-Whitney test.
